# CD38-specific nanobody-based bispecific antibody recruiters (BARs) redirect complement-dependent cytotoxicity toward multiple myeloma cells

**DOI:** 10.1038/s41598-025-25194-y

**Published:** 2025-11-18

**Authors:** Luca Julius Pape, Anna Josephine Gebhardt, Marten Dannenberg, Henry Risch, Anya Duttmann, Katja Weisel, Julia Hambach, Friedrich Koch-Nolte, Peter Bannas

**Affiliations:** 1 Department of Diagnostic and Interventional Radiology and Nuclear Medicine, University Medical Center Hamburg-Eppendorf, Hamburg, Germany; 2 Institute of Immunology, University Medical Center Hamburg-Eppendorf, Hamburg, Germany; 3 Department of Oncology, Hematology and Bone Marrow Transplantation, University Medical Center Hamburg-Eppendorf, Hamburg, Germany

**Keywords:** CD38, Multiple myeloma, Bispecific engagers, Antibody recruiting molecules, Nanobodies, Complement-dependent cytotoxicity, Targeted therapies, Antibody fragment therapy, Targeted therapies

## Abstract

Bispecific antibody recruiters (BARs) are an innovative class of immunotherapeutics that redirect endogenous antibodies to tumor cells. BARs comprise a tumor-binding and an antibody-binding module, enabling endogenous antibodies to opsonize tumor cells and induce F_C_-dependent effector functions such as complement-dependent cytotoxicity (CDC). CD38 is a validated target for myeloma therapy, as evidenced by the success of CD38-specific monoclonal antibodies daratumumab and isatuximab. Nanobodies are single variable immunoglobulin domains derived from camelid heavy chain antibodies. Here, we report the generation of nanobody-based BARs recognizing CD38 and assess their cytotoxicity against CD38-expressing myeloma cells in vitro and ex vivo. We constructed three CD38-specific BARs recognizing distinct, non-overlapping CD38 epitopes (E1-BAR, E2-BAR, and E3-BAR) by genetically fusing CD38-specific nanobodies to a human immunoglobulin κ light chain-specific nanobody. All BARs exhibited simultaneous binding to CD38 and IgGκ. Nonlinear regression revealed EC_50_ values of 0.73 nM (E1-BAR), 0.21 nM (E2-BAR), and 0.97 nM (E3-BAR). In patient-derived myeloma cells, E1-BAR reduced viability to 29 ± 18%, while E2-BAR and E3-BAR achieved 62 ± 49% and 57 ± 41%, respectively. In vivo, BAR half-life was markedly increased by IgGκ binding. Our results demonstrate the feasibility of CD38-specific nanobody-based BARs as therapeutics for multiple myeloma.

**Supplementary Information:**

The online version contains supplementary material available at 10.1038/s41598-025-25194-y.

## Introduction

Multiple myeloma (MM) is a malignant plasma cell disorder characterized by clonal expansion of aberrant plasma cells^1^. MM is one of the most frequent hematological malignancies with a global age-standardized incidence rate of 1.79 per 100,000 people, accounting for 1.1% of all cancer deaths^1,2^. Despite recent improvements in overall survival rates, most patients eventually exhibit treatment resistance and relapse, highlighting the need for additional treatment options^2–4^. CD38 is a major target for immunotherapy of myeloma, as illustrated by the clinical success of the CD38-specific monoclonal antibodies (mAbs) daratumumab and isatuximab^5,6^.

To improve therapeutic efficacy, antibody development has transitioned from conventional antibodies to more complex antibody-based constructs. This includes innovations such as biparatopic and bispecific antibodies^7,8^, chimeric antigen receptors (CARs)^9,10^, bispecific T- or NK cell engagers (BiTEs/BiKEs)^11,12^, or bispecific antibody recruiters (BARs)^13,14^.

BARs are an innovative class of immunotherapeutics that redirect endogenous antibodies to desired targets, such as tumor cells, bacteria, or virally infected cells^13–18^. Current BAR designs for tumor therapy consist of an antibody-binding module and a tumor-binding module^14^. Antibodies recruited to the tumor cell surface then induce F_C_-dependent effector functions such as complement-dependent cytotoxicity (CDC) or antibody-dependent cellular cytotoxicity (ADCC).

Nanobodies are single variable immunoglobulin domains that can be isolated from camelid heavy chain antibodies^19,20^. They demonstrate high thermal stability^21,22^ and a tendency to refold after denaturation^23^. Their high solubility^20^, low immunogenicity^20^, and ease of reformatting^24^ make them ideally suited as interchangeable building blocks of recombinant antibody constructs such as BARs.

The aim of our study was to develop CD38-specific nanobody-based BARs specific for three distinct, non-overlapping epitopes on CD38 and to evaluate their potential to induce lysis of myeloma cells in vitro and ex vivo.

## Results

### Nanobody-based CD38-specific BARs specifically and simultaneously bind to CD38 and human IgGκ

**Fig. 1:**
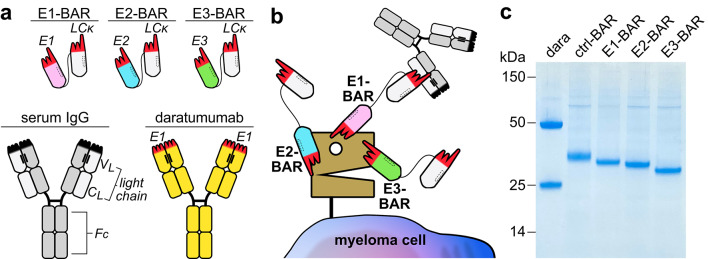
Structure, binding sites, and purity of three nanobody-based CD38-specific bispecific antibody recruiters (BARs). (**a**) Schematic illustration of different antibody constructs targeting CD38. The top row shows three nanobody-based BARs (30 kDa), each consisting of two nanobodies fused via a glycine-serine linker. The N-terminal nanobody in each of the three BARs recognizes one of three distinct epitopes on CD38: E1, pink; E2, blue; E3, green. The C-terminal nanobody is specific for human immunoglobulin kappa light chain (LC_κ_, white). The bottom row shows serum IgGκ (grey, left) and the conventional CD38-specific monoclonal human IgG1κ antibody daratumumab (yellow, right). Immunoglobulin light chain variable domain (V_L_), constant domain (C_L_), crystallizable fragment (F_C_), and targeted epitopes (E1–E3) are indicated in italics. (**b**) Schematic illustration of the binding sites of the three CD38-specific BARs. E1-BAR recognizes an epitope that overlaps with the epitope bound by daratumumab (E1). Both E2-BAR and E3-BAR bind distinct, independent epitopes on CD38 (E2, E3). CD38-specific BARs recruit serum IgGκ (grey) to CD38-expressing myeloma cells. The C-terminal nanobody (white) recognizes human kappa light chain while the N-terminal nanobody (pink, blue, or green) recognizes one of three epitopes on CD38. The interaction between serum IgGκ and CD38-BARs is illustrated using E1-BAR as a representative example. (**c**) Coomassie staining of an SDS-PAGE under reducing conditions with HEK cell supernatants of control-BAR, CD38-specific BARs (10 µL/lane), and purified daratumumab (1 µg/lane). The original uncropped gel is presented in Supplementary Figure S1.

**Fig. 2:**
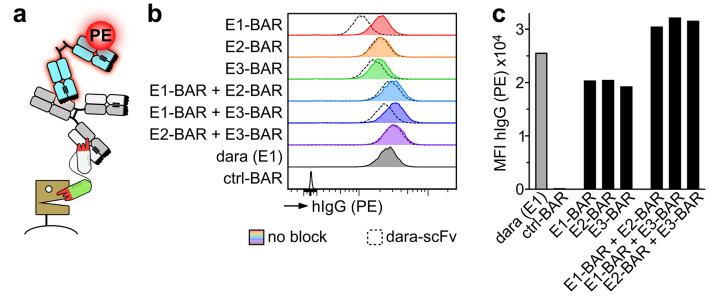
Nanobody-based CD38-specific BARs show specific and simultaneous binding to both CD38 and kappa light chain of human IgG. (**a**) Schematic illustration of the experimental strategy to verify binding of BARs to both CD38 and kappa light chain of human IgG. CD38-transfected HEK cells were incubated with or without 100 nM dara-scFv to block epitope E1 of CD38 (not shown). Cells were then incubated with BARs for 20 min. Human pooled serum was added as a source of unspecific IgGκ antibodies (grey). Simultaneous binding of the BARs to CD38 and IgGκ was detected using a PE-conjugated F(ab’)2 antibody (cyan) specific for human IgG. (**b**) FACS histograms showing simultaneous binding of CD38-BARs to both CD38 and kappa light chain of human IgG without blocking (colored histograms) and after blocking with 100 nM dara-scFv (dashed histograms). Daratumumab was used as a positive control (grey histogram). HEK cells incubated with unspecific ctrl-BAR (open histogram) were used as a negative control. (**c**) Median fluorescent intensities of detected hIgG (PE) as an indicator of IgG recruitment. Daratumumab served as a positive control. ctrl-BAR served as a negative control.

We generated three nanobody-based CD38-specific BARs, designated E1-BAR, E2-BAR, and E3-BAR (Fig. 1). All BARs were readily produced in HEK cells without forming aggregates (Supplementary Fig. S2, Supplementary Fig. S3). Specific binding of CD38-BARs to CD38 was analyzed using HEK293-T cells stably transfected with human CD38 (Fig. 2). Cells were incubated with or without dara-scFv to assess competitive binding to epitope E1 of CD38. Cells were then incubated with either single CD38-BARs, a combination of two CD38-BARs, ctrl-BAR, or without a BAR. 25% human pooled serum was added as a source of κ light chains. Daratumumab served as a positive control. Binding was detected using a PE-conjugated detection antibody specific for human IgG. This strategy allowed us to determine specific and simultaneous binding of the BARs to both CD38 and κ light chain. All three CD38-BARs (E1-BAR, E2-BAR, and E3-BAR) showed simultaneous binding to κ light chain and CD38-expressing HEK293-T cells irrespective of their epitope specificity (E1, E2, or E3) or combination with another CD38-BAR. The ctrl-BAR did not bind the cells, confirming the specific binding of the CD38-BARs to CD38-expressing cells via their respective CD38-specific nanobody. Daratumumab showed strong binding, confirming high CD38 expression of the target cells.

Blocking with dara-scFv strongly decreased binding of E1-BAR and mildly decreased binding of E3-BAR and the combinations of E1-BAR + E2-BAR and E1-BAR + E3-BAR (Fig. 2b). No effect was observed on the binding of E2-BAR or the combination of E2-BAR + E3-BAR.

Median fluorescent intensities (MFIs) of hIgG (PE) were compared to quantify the amount of IgG recruited by the BAR-treated groups. MFIs in cells treated with a single CD38-BAR were lower than in cells treated with a combination of two BARs, indicating that a combination of two BARs increases the amount of IgG recruited to the cell.

In summary, all three CD38-BARs specifically and simultaneously bound to human CD38 and human κ light chain. Combinations of two BARs enhanced IgG recruitment to target cells compared to single constructs.

### CD38-specific BARs mediate dose- and time-dependent CDC of tumor cell lines in vitro

**Fig. 3:**
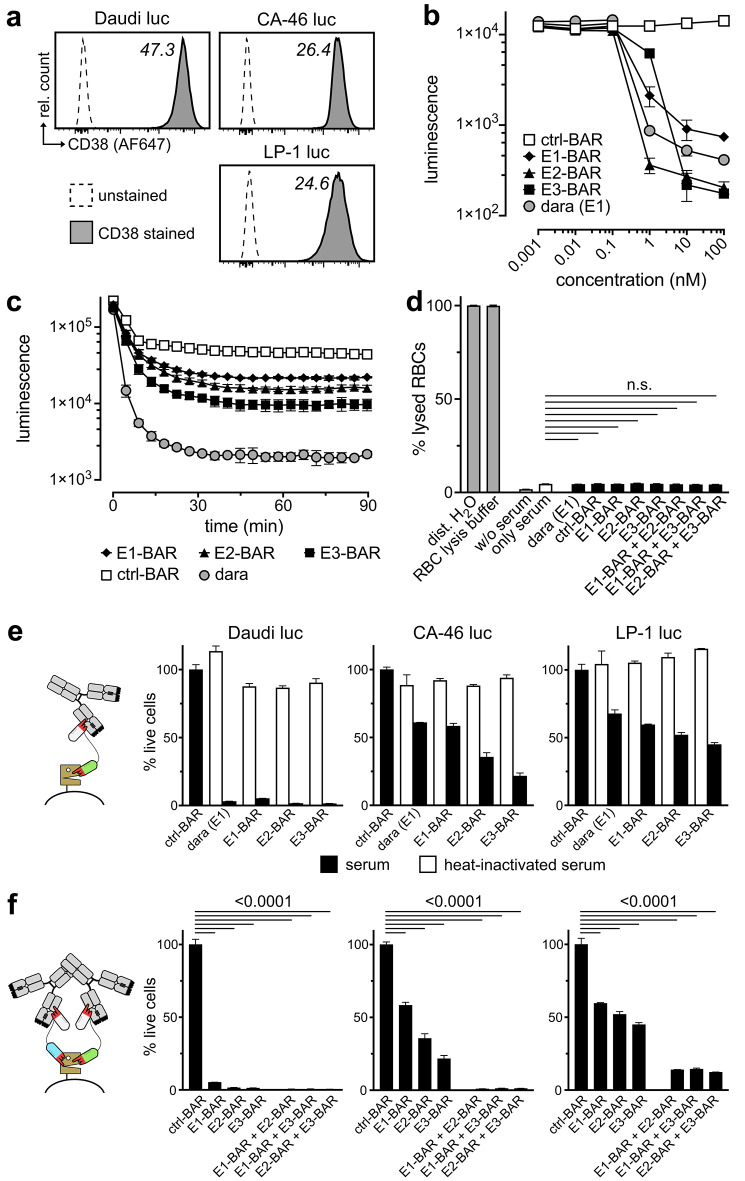
Nanobody-based CD38-specific BARs induce dose- and time-dependent complement-dependent cytotoxicity (CDC) against CD38-expressing cell lines in vitro. (**a**) Cell surface expression of CD38 on Daudi luc, CA-46 luc, and LP-1 luc cells. Median fluorescent intensity (× 10^3^) of CD38 is indicated next to the histograms. (**b**) Viability of Daudi luc cells after incubation with 0.001, 0.01, 0.1, 1, 10, and 100 nM of CD38-specific BARs, control-BAR (ctrl-BAR), or daratumumab in the presence of 25% human pooled serum. Bioluminescence was measured as an indicator of cell viability. (**c**) Time-resolved viability of Daudi luc cells incubated with 100 nM of CD38-BARs, ctrl-BAR, or daratumumab 0–90 min after the addition of 25% human pooled serum. (**d**) Lysis of human erythrocytes (RBCs) after incubation with 100 nM of CD38-BARs, ctrl-BAR, and daratumumab in the presence of 25% human pooled serum. OD_405_ was measured as an indicator of hemolysis. Distilled H_2_O and RBC lysis buffer served as positive controls. (**e**) Viability of Daudi luc, CA-46 luc, and LP-1 luc cells after incubation with 100 nM of individual CD38-specific BARs or daratumumab. Active human pooled serum served as a source of endogenous antibodies and complement (black bars). Heat-inactivated serum was used as negative control (white bars). (**f**) Viability of the same cell lines after incubation with combinations (50 + 50 nM) of two BARs targeting distinct, non-overlapping epitopes on CD38. Individual BARs targeting a single epitope of CD38 or an irrelevant antigen (ctrl-BAR) were used as controls. All diagrams show means ± standard deviation (SD) of three parallel replicates. Results of two-way ANOVA between serum and BARs (**d**) or ctrl-BAR and BARs (**f**) are indicated as *p*-values above the diagrams. N.s. indicates no significant difference, i.e., *p* ≥ 0.05.

The capacity of CD38-specific BARs to induce complement-dependent cytotoxicity (CDC) was evaluated using Daudi luc, CA-46 luc, and LP-1 luc tumor cell lines as targets. All cell lines showed high and uniform cell surface levels of CD38 (Fig. 3a).

All three CD38-specific BARs induced dose-dependent and time-dependent CDC against tumor cells (Fig. 3b,c). Nonlinear regression revealed EC_50_ values of 0.73 nM for E1-BAR, 0.21 nM for E2-BAR, and 0.97 nM for E3-BAR. EC_50_ for daratumumab was 0.63 nM. The EC_50_ corresponds to the BAR concentration required to induce 50% CDC compared to the ctrl-BAR. None of the BARs mediated CDC against human erythrocytes (Fig. 3d). The three BARs differed in their potency to induce CDC (Fig. 3e): E1-BAR consistently induced weaker CDC than E2-BAR (*p* < 0.05 across all cell lines) and E3-BAR (*p* < 0.01 across all cell lines). E2-BAR induced weaker CDC than E3-BAR in CA-46 (*p* < 0.0001) and LP-1 cells (*p* = 0.0028), but not in Daudi cells (*p* > 0.05). Compared to daratumumab, E1-BAR induced stronger CDC in LP-1 cells (*p* = 0.0008), but not in CA-46 or Daudi cells (*p* > 0.05), while both E2-BAR and E3-BAR showed stronger activity in LP-1 and CA-46 cells (both *p* < 0.0001), but not in Daudi cells (*p* > 0.05). The degree of CDC induction correlated with CD38 expression levels. Controls using heat-inactivated serum revealed complement dependency of cytolysis.

Next, we analyzed the potential of combining two BARs targeting distinct, non-overlapping epitopes on CD38 to induce CDC (Fig. 3f). To detect synergistic effects, we used half the concentration of each BAR in the combinations compared to the previous assay. All three combinations (E1-BAR + E2-BAR, E1-BAR + E3-BAR, and E2-BAR + E3-BAR) were stronger than single agents and mediated superior CDC compared to ctrl-BAR (*p* < 0.0001 across all cell lines) and daratumumab (*p* < 0.0001 for CA-46 and LP-1, *p* > 0.05 for Daudi).

By contrast, neither CD38-BARs nor daratumumab mediated CDC against RPMI-8226 luc and OPM-2 luc cells, which express high levels of the complement-inhibitory proteins CD55 and CD59 (Supplementary Figure S5).

In summary, all three CD38-specific BARs consistently induced CDC in susceptible cells lines in vitro, with combinations of two BARs markedly enhancing cytotoxicity.

### CD38-specific BARs mediate moderate ADCC of tumor cell lines in vitro

**Fig. 4:**
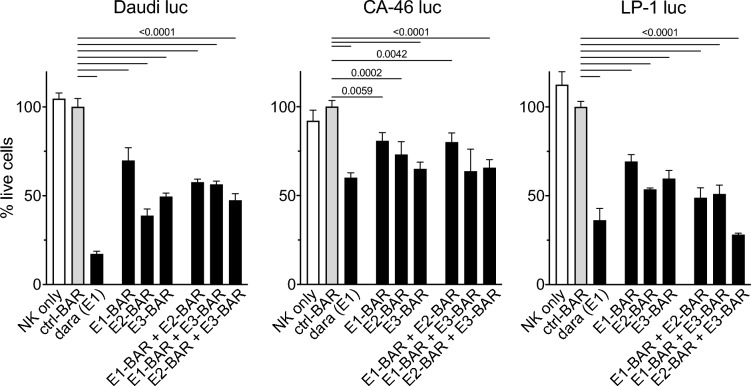
Nanobody-based CD38-specific BARs induce antibody-dependent cellular cytotoxicity (ADCC) against CD38-expressing cell lines in vitro. Viability of Daudi luc (left), CA-46 luc (middle), and LP-1 luc cells (right) after incubation with 100 nM of CD38-BARs. NK92 cells were added at an effector-to-target ratio of 3:1 and incubated with tumor cells for 180 min. 25% inactivated human pooled serum was used as a source of antibodies. Daratumumab served as a positive control. Ctrl-BAR and cells incubated without BAR served as negative controls. Depicted are means ± SD of three parallel replicates. Results of a two-way ANOVA between ctrl-BAR and BARs are indicated as *p*-values above the diagrams.

The capacity of CD38-specific BARs to induce antibody-dependent cellular cytotoxicity (ADCC) was evaluated using Daudi luc, CA-46 luc, and LP-1 luc tumor cell lines as targets and hCD16-transduced NK92 cells as effectors (Fig. 4). All three CD38-specific BARs mediated moderate ADCC against all cell lines. No increase in ADCC induction was observed when combining two BARs targeting distinct epitopes of CD38. Daratumumab induced moderate to high ADCC, confirming susceptibility of the cell lines to ADCC.

In summary, all three CD38-specific BARs induced moderate ADCC in CD38-expressing cell lines in vitro.

### CD38-specific BARs mediate effective CDC of primary myeloma cells ex vivo

**Fig. 5:**
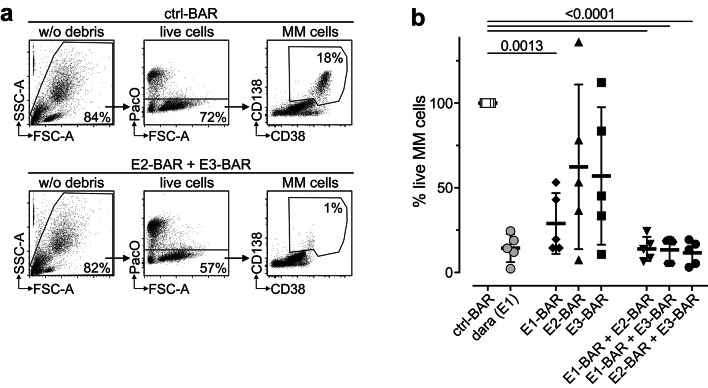
The combination of two CD38-specific BARs induces strong complement-dependent cytotoxicity against primary myeloma cells from patient biopsies ex vivo. Bone marrow mononuclear cells from myeloma patients (n = 5) were incubated with CD38-BARs alone or with the combination of two CD38-BARs. Daratumumab (dara) served as a positive control. A ctrl-BAR targeting an irrelevant antigen served as a negative control. Human pooled serum (25%) was used as a source of complement and of polyclonal, unspecific antibodies. Induction of CDC was quantified by flow cytometric analysis using counting beads and the viability dye Pacific Orange. (**a**) Gating was carried out on live cells by excluding beads and cellular debris (left panels), doublets (not shown), and dead cells (middle panels). Myeloma cells were identified by their strong expression of CD38 and CD138 (right panels). Numbers indicate the percentage of cells included in the respective gates. (**b**) Absolute numbers of surviving myeloma cells (PacO-/CD38 + /CD138 +) were calculated using cell counting beads. Percentages of live myeloma cells were determined relative to samples incubated with the ctrl-BAR (set to 100%). Data represent mean ± SD. One-way ANOVA was performed to detect statistically significant differences compared to the ctrl-BAR.

The capacity of CD38-specific BARs to induce CDC ex vivo was tested on fresh bone marrow cells from five multiple myeloma patients (Fig. 5).

E1-BAR induced moderate to strong CDC of MM cells in all cases (viability: 28.9 ± 17.9%). E2-BAR and E3-BAR induced varying degrees of CDC (viability: 62.4 ± 48.6% and 57.0 ± 40.6%, respectively).

All three combinations of two BARs targeting distinct epitopes on CD38 mediated strong and comparable CDC in all patients. CDC induction by E1-BAR + E2-BAR (viability: 13.9 ± 7.1%), E1-BAR + E3-BAR (viability: 13.4 ± 7.3%) and E2-BAR + E3-BAR (viability: 11.6 ± 7.1%) was comparable to CDC induction by daratumumab (viability: 14.4 ± 8.3%), as determined by a statistically non-significant difference via two-way ANOVA (*p* > 0.999).

Ctrl-BAR was used as a negative control (viability normalized to 100%) while daratumumab was used as a positive control.

In summary, the combination of two CD38-specific BARs targeting distinct epitopes induced strong CDC against primary human myeloma cells ex vivo.

### CD38-specific BARs bind to IgGκ in vivo, effectively increasing their half-life

**Fig. 6:**
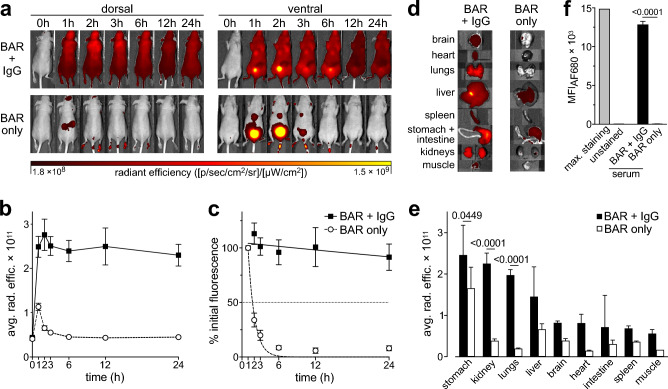
CD38-specific BARs bind to IgGκ in vivo, prolonging their serum half-life. NMRI-nude mice were randomly assigned to receive tail vein injections of AF680-conjugated E3-BAR (BAR only, n = 4) or AF680-conjugated E3-BAR 1 h after administration of IgGκ (BAR + IgG, n = 4). Fluorescent signals were monitored for 24 h post injection of the BAR. (**a**) Fluorescent signals seen from the dorsal (left) or ventral side (right) of one representative mouse per treatment group. Images are equally levelled to allow fair and direct visual comparison. (**b**) Equally sized rectangular whole-body regions of interest (ROIs) were drawn around each mouse to obtain dorsal whole-body fluorescent levels as a measure of remaining BAR concentration. (**c**) ROI data was normalized to the first time point after injection to allow the determination of whole-body half-life. (**d**) Ex vivo fluorescence of organs explanted at the 24 h time point. Depicted are organs of one representative mouse per treatment group. (**e**) Circular ROIs were drawn inside organs to obtain organ-specific fluorescent levels as an indicator of biodistribution. (**f**) Serum obtained at the 24 h time point was used to stain CD38-expressing LP-1 luc cells followed by flow cytometric analysis. Cells stained in vitro using the same AF680-conjugated E3-BAR served as a positive control. Unstained cells served as a negative control. All diagrams depict means ± SD. Results of a two-way ANOVA between the BAR only and BAR + IgG group are indicated as *p*-values above the bar charts when the difference was statistically significant (*p* < 0.05).

The half-life and IgG binding capacity of CD38-specific BARs were assessed in a mouse model in vivo (Fig. 6). NMRI-nude mice were randomly assigned to receive either AF680-conjugated E3-BAR alone (BAR only, n = 4) or AF680-conjugated E3-BAR after administration of IgGκ (BAR + IgG, n = 4).

In the BAR + IgG group, dorsal whole-body optical imaging over 24 h showed strong, widespread signals at all time points (Fig. 6a). In contrast, the BAR only group showed signals only around the kidneys at 1 h, consistent with rapid renal clearance. Ventral imaging in the BAR + IgG group also showed strong whole-body signals with peaks near the bladder, suggesting some renal filtration of unbound BARs. In the BAR only group, strong signals were visible at 1–3 h with peaks around the bladder and lower thorax.

ROI analysis confirmed these trends: dorsal whole-body fluorescence in the BAR + IgG group decreased slowly over 24 h, whereas fluorescence in the BAR only group decreased rapidly until 6 h (Fig. 6b).

Whole-body half-life was calculated from a linear regression based on fluorescence normalized to the 1 h time point (Fig. 6c). Half-life was 44 min (95% CI: 26–72 min, R^2^: 0.97 in the BAR only group) and 125 h (95% CI: 45 h–undetermined, R^2^: 0.44) in the BAR + IgG group. The half-life value for the BAR + IgG group must be interpreted with caution, as fluorescence decay within the 24 h experimental window was minimal, resulting in a limited curve fit.

Biodistribution was assessed from organs explanted at 24 h (Fig. 6d). The BAR + IgG group revealed strong signals in the stomach, kidneys, lungs, and liver. In the BAR only group, signals were observed in the stomach and liver. ROI analysis of the organs confirmed this observation (Fig. 6e). Median fluorescence intensity of LP-1 cells stained with serum from the BAR + IgG group was significantly higher than in the BAR only group, confirming markedly higher serum concentrations at the 24 h time point (Fig. 6f).

In summary, binding to IgGκ markedly prolonged the in vivo half-life of CD38-specific BARs.

## Discussion

Our results demonstrate the feasibility of using CD38-specific nanobody-based BARs to effectively kill myeloma cells in vitro and ex vivo. Specifically, three BARs recognizing distinct, non-overlapping epitopes on human CD38 induced dose- and time-dependent CDC against tumor cell lines in vitro. The combination of two BARs targeting different epitopes on CD38 dramatically enhanced the CDC against tumor cell lines in vitro and primary MM cells ex vivo.

All three CD38-BARs were readily produced in HEK cells and bound specifically and simultaneously to human cell surface CD38 and serum immunoglobulin κ light chain. The combination of two CD38-BARs increased the amount of IgG recruited to the cell compared to a single BAR. When used as single agents in combination with human serum, all CD38-BARs mediated dose- and time-dependent CDC. Combining two CD38-specific BARs targeting distinct epitopes resulted in a strong increase in CDC. This likely reflects more efficient hexamerization of IgG simultaneously recruited by two BARs, promoting C1q engagement and complement activation^25–27^. Single BARs engage only one IgG, likely inducing less efficient hexamerization. CDC induction was greatest against Daudi luc cells, moderate against CA-46 luc cells, and weakest against LP-1 luc cells, suggesting that higher levels of CD38 expression permit stronger CDC induction. No CDC was observed against erythrocytes or in cell lines expressing high levels of the complement-inhibitory proteins CD55 and CD59, consistent with published findings^28^. In patient-derived samples, E1-BAR induced moderate to strong CDC while E2- and E3-BAR showed variable activity, suggesting that the targeted epitope on CD38 affects complement activation^29,30^. Since CD38 is often blocked by daratumumab in clinical practice^31^, and dara-scFv blocking variably decreased binding of all BARs except E2-BAR and E2-BAR + E3-BAR, further development should focus on these candidates as potential supplementary therapeutics alongside daratumumab. Importantly, the BAR concentration used in this study (100 nM) is far below the serum concentrations achieved during daratumumab treatment (maximum c_trough_ 593 µg/mL, i.e. ~ 4 µM after subcutaneous administration), supporting the potential for clinical translation^32^.

ADCC induction in vitro was moderate and comparable across all BARs. No difference was observed between single BARs and the combination of two BARs, contrasting with CDC results. This likely reflects a less pronounced role of F_C_-F_C_ interactions on effector cell activation during ADCC compared to C1q recruitment in CDC, consistent with previous studies using CDC-optimized antibodies^27,33^.

Our nanobody-based BARs possess several advantages compared to existing antibody recruiting molecules described in the literature^14^. Many current antibody recruiting molecules rely on common non-self antigens such as L-rhamnose (L-Rha)^34–38^ or dinitrophenol (DNP)^39–43^ as antibody-binding modules to recruit endogenous antibodies.

By design, these can only recruit the limited pool of pre-existing endogenous anti-L-Rha or anti-DNP antibodies and rely on previous exposure to the antigen. In contrast, our nanobody-based CD38-specific BARs use a human κ light chain-specific nanobody to recruit all κ light chain antibodies irrespective of their specificity. They are able to draw from a larger and therefore more diverse pool of endogenous antibodies with different glycosylation variants^44,45^ and subclasses^46,47^, potentially mediating more effective complement recruitment. Κ light chain was chosen as a target since intact IgGκ is, on average, more abundant in human serum than IgGλ^48^. As class switching from IgM to IgG, IgE or IgA affects only heavy chains, it is conceivable that other immunoglobulin subclasses with κ light chains would also be recruited by our BARs^49^.

The dependence of our BARs on κ light chain can be a potential disadvantage compared to existing BAR constructs using other antibody-binding modules. Since the κ/λ ratio in serum differs between individuals and can shift in disease states (e.g. autoimmune disease^50^ or B cell malignancies^51^), our constructs would potentially show more variable efficacy compared to F_C_-binding designs. Additionally, circulating free κ light chains might compete with intact IgGκ binding in multiple myeloma patients.

A related concept that highlights the advantage of nanobodies as complement-activating tumor therapeutics has recently been described by Pedersen et al. In their work, they developed a bispecific complement engager (BiCE) that induced CDC against CD38-expressing tumor cells^52^. To this end, a nanobody specific for complement protein C1q was genetically fused a to a nanobody specific for CD38. Similarly to our CD38-BARs, the CD38-specific BiCE mediated a strong CDC against CD38-expressing tumor cells. Future studies are needed to allow side-by-side comparison of CDC induction by BARs and BiCE. Since the role of the complement system in cancer therapy is subject to debate^25,53–55^, future studies should also analyze the capacity of these constructs to induce additional cytotoxic effector mechanisms, such as antibody-dependent cellular phagocytosis (ADCP).

An inherent disadvantage of many nanobody-based constructs is their short serum half-life, owing to rapid renal filtration^20,56–59^. However, as the CD38-BARs bind to κ light chains, we hypothesized that binding to circulating antibodies would increase their half-life. A similar approach to prolong serum half-life has been reported for other BARs^17,60^. Using fluorochrome-conjugated E3-BAR as a representative candidate, we confirmed a markedly increased half-life in mice receiving BAR + IgG compared with BAR only.

The combination of two BARs targeting distinct epitopes of CD38 dramatically enhanced the CDC and showed potent killing across all cell lines in vitro and primary MM cells ex vivo. These results indicate that biparatopic targeting of CD38 holds promise for improved CDC induction in future studies.

A central advantage of nanobodies as easily interchangeable tumor binding modules is the option to generate biparatopic CD38-specific BARs by combining two CD38-specific nanobodies in a single BAR. As epitope E1 is often blocked by daratumumab in clinical practice^31^, further development should focus on the two candidates targeting epitopes E2 and E3, which are distinct from the epitope bound by daratumumab^61,62^.

Our nanobody-based constructs further enable the generation of bispecific BARs by combining two nanobodies specific for two different tumor antigens. Ciltacabtagene autoleucel (cilta-cel), the first FDA- and EMA-approved nanobody-based chimeric antigen receptor, has shown impressive outcomes in multiple myeloma^63,64^. Tumor binding in cilta-cel is mediated via two nanobodies specific for different epitopes on B-cell maturation antigen (BCMA)^63,65^. To enable targeting of heterogeneous tumor cell populations, future studies using a bispecific BAR consisting of one of cilta-cel’s BCMA-specific nanobodies and one CD38-specific nanobody would be of interest. Furthermore, targeting two antigens could potentially address limitations in CDC efficacy of our constructs, as CD38 expression is known to decrease under daratumumab therapy^28^, at least partly due to internalization^49^.

In conclusion, we have demonstrated the feasibility of using nanobody-based, CD38-specific BARs to kill hematological tumor cells in vitro and ex vivo. Given their potent CDC induction and versatility, nanobody-based CD38-specific BARs warrant further in vivo validation and development as therapeutics for multiple myeloma.

## Materials and methods

### Cell lines

Three human multiple myeloma cell lines (LP-1, RPMI-8226, and OPM-2), two human Burkitt lymphoma cell lines (CA-46 and Daudi), HEK293-T, and NK92 cells were obtained from the German Collection of Microorganisms and Cell Culture (DSMZ, Braunschweig, Germany). HEK293-6E cells were licensed from the National Research Council, Canada. Lentiviral transduction was used to generate LP-1 luc, RPMI-8226 luc, OPM-2 luc, CA-46 luc, and Daudi luc cell lines stably expressing GFP and the luc2 variant of *Photinus pyralis* luciferase (Promega, Madison, WI, USA) as described previously^66,67^. Transduced cells were selected by FACS and subsequent propagation in culture medium containing 1 µg/mL puromycin. Expression of GFP and luciferase was monitored regularly by flow cytometry and luciferase-based luminescence assays. Cell lines were cultured in RPMI 1640 medium (Gibco, Thermo Fisher, Waltham, MA, USA) supplemented with 5% (v/v) fetal bovine serum (Gibco), 2 mM L-glutamine (Gibco), and 2 mM sodium pyruvate (Gibco). Cell surface expression of human CD38, CD55, and CD59 on tumor cell lines was evaluated via flow cytometry after incubating cells with 1:500 (v/v) CD38-specific nanobody JK36-AF647^62^, CD55-specific antibody IA10 (cat. #555,694, Becton Dickinson, Franklin Lakes, NJ, USA), or CD59-specific antibody p282 (cat. #555,764, Becton Dickinson) for 20 min at 4 °C.

Stable expression of human CD38 in HEK293-T cells was achieved by polyethylenimine-mediated transfection with cDNA expression vectors. Transfected cells were selected by FACS and subsequent propagation in DMEM culture medium (Gibco) containing 1 µg/mL puromycin, 5% (v/v) fetal bovine serum (Gibco), 2 mM L-glutamine (Gibco), and 2 mM sodium pyruvate (Gibco).

Stable expression of high levels of human CD16 and eGFP in NK92 cells was achieved by retroviral transduction using the sF91 vector. The sequence for human CD16 was kindly provided by Béatrice Clémenceau, Nantes, France^68^. To prevent CD38-dependent fratricide, the gene encoding CD38 was inactivated by CRISPR/Cas9 (cat. #sc-401117-NIC, Santa Cruz Biotechnology, Dallas, TX, USA). NK92 cells were kept in alpha-MEM culture medium (Gibco) supplemented with 10% horse serum (Gibco), 5 mM L-glutamine (Gibco), and 5 ng/mL IL-2 (Proleukin, Novartis, Nürnberg, Germany).

### CD38-specific BARs and conventional antibodies

Human CD38-specific nanobodies WF211, MU1067, and JK36 were generated from immunized llamas as described previously^62,69^. WF211 binds epitope 1 (E1), MU1067 binds epitope 2 (E2), and JK36 binds epitope 3 (E3) of human CD38^62,70^.

Nanobody L-15 binds *Clostridium difficile* Toxin A and was used as a negative control (ctrl) in all experiments^71^. The sequence for the human κ light chain-specific nanobody Hu-kappa-1 was obtained from patent WO 2006/059904.

To improve clarity, the generated BARs containing the three different CD38-specific nanobodies (WF211 (E1), MU1067 (E2), JK36 (E3)) were designated E1-BAR, E2-BAR, and E3-BAR (Fig. 1a, b). E1-BAR, E2-BAR, E3-BAR, and ctrl-BAR were generated by subcloning the coding region of the respective CD38-specific or control nanobody upstream of the coding region for Hu-kappa-1. These were linked by a flexible 15 amino acid glycine-serine linker and cloned into the pCSE2.5 vector (kindly provided by Thomas Schirrmann, Braunschweig, Germany) using NcoI/Not1 (CD38-specific nanobodies) and Not1/Xba1 (Hu-kappa-1) restriction sites. The amino acid sequences for all BARs are reported in Supplementary Tab. S6. The respective off-rates (k_diss_) or affinities (K_d_) of the individual nanobodies have been published previously: WF211 4.5 × 10^–3^ s^-1^, MU1067 1.2 × 10^–4^ s^-1^, JK36 2.0 × 10^–4^ s^-1^, L-15 1.5 nM, and Hu-kappa-1 7.8 × 10^–4^ s^-1^^62,71,72^. It is reasonable to assume that off-rates or affinities are largely retained in our BAR format, since the flexible glycine-serine linker preserves independent folding and binding of each domain^73^.

BARs were expressed in transiently transfected HEK293-6E cells cultivated in serum-free medium. Supernatants were harvested six days after transfection and cleared by centrifugation. Concentration of antibody constructs were assessed by reducing SDS-PAGE and InstantBlue™ Coomassie staining (Fig. 1c). A non-reducing SDS-PAGE was performed to assess purity (Supplementary Fig. S2). To assess potential protein aggregation, supernatants of CD38-BARs or ctrl-BAR that had been stored at 4 °C for several weeks were centrifuged for 10 min at 4350 g using Amicon Ultra centrifugal filters with a 100 kDa molecular weight cut-off. Flow-through and retained fractions were collected separately and analyzed by non-reducing SDS-PAGE (Supplementary Fig. S3).

Daratumumab (Darzalex) was purchased from Janssen-Cilag, Neuss, Germany.

### Binding analyses

Binding analyses of CD38-specific BARs and ctrl-BAR were performed on HEK293-T cells stably transfected with human CD38. HEK cells were chosen since human lymphoma and myeloma cell lines had shown surface expression of IgG in previous experiments, interfering with the staining of recruited IgG. To detect the competitive effect of daratumumab on BAR binding, cells were incubated with or without 100 nM single-chain variable fragment (scFv) of daratumumab for 20 min. Cells were then incubated with 10 µL of CD38-BARs, ctrl-BAR, or without any antibody at 4 °C for 20 min in 100 µL of PBS + 0.2% BSA. 100 nM daratumumab was used as a positive control. 25% human pooled serum was then added as a source of polyclonal human IgGκ antibodies and cells were incubated at 4 °C for an additional 20 min. Cells were then washed and bound antibodies were detected with a PE-conjugated, human IgG-specific donkey IgG F(ab’)2 detection antibody (cat. #SEC-183768, Dianova, Hamburg, Germany). Cells were washed twice before flow cytometric analysis (FACS Canto II, Becton Dickinson). Data was analyzed using FlowJo v10 (Becton Dickinson). The gating strategy is visualized in Supplementary Fig. S4.

### CDC of tumor cell lines and human erythrocytes

Daudi luc, CA-46 luc, and LP-1 luc tumor cell lines were incubated in 100 µL PBS + 0.2% BSA containing 10 µL supernatant (~ 100 nM) or serial dilutions of E1-BAR, E2-BAR, E3-BAR, or ctrl-BAR at 37 °C for 20 min. When two antibody constructs were combined (BAR + BAR), only 5 µL supernatant of each construct was used. 100 nM daratumumab served as a positive control. 25% human pooled serum was added as a source of complement and non-specific antibodies before further incubation at 37 °C for an additional 90 min. Heat-inactivated human pooled serum (30 min at 56 °C) was used as a negative control. Cells were washed once and resuspended in PBS + 0.2% BSA containing 37.5 µg/mL D-luciferin (Biosynth, Staad, Switzerland). Bioluminescence was measured after 25 min at 37 °C using a microplate reader (Victor^3^, PerkinElmer, Waltham, MA, USA). The degree of CDC induction was calculated as follows:

$$\% \,\text{live cells}=\frac{\text{BLI signal [sample]}}{\text{BLI signal [ctrl-BAR]}} \times 100 \%$$

To evaluate CDC kinetics, Daudi luc cells were incubated with BARs and controls as described above. Cells were incubated in 50 µg/mL D-luciferin for 20 min at 37 °C to reach peak luminescence before the addition of 25% human pooled serum. Luminescence was then measured 1/min for 90 min starting 60 s after the addition of serum.

Hemolysis of human erythrocytes was evaluated as described previously^74^. Briefly, pooled human erythrocytes (RBCs) were incubated with BARs as detailed above. 25% human pooled serum was added and cells were incubated for 90 min at 37 °C. Samples incubated with distilled water or RBC lysis buffer (NH_4_Cl + KHCO_3_ + EDTA) served as positive controls. Samples incubated without BARs or without serum served as negative controls. Optical density (OD) at 405 nm was measured using a microplate reader (Victor^3^) and the degree of hemolysis was calculated as follows:

$$\%\text{ lysed RBCs}=\frac{\text{OD405 [sample]}}{\text{OD405 [dist. }{\text{H}}_{2}{\text{O}}\text{]}} \times 100 \%$$

### ADCC of tumor cell lines

Daudi luc, CA-46 luc, and LP-1 luc tumor cell lines were incubated in alpha-MEM medium with or without CD38-specific BARs, ctrl-BAR, or daratumumab as described above. 25% heat-inactivated human pooled serum was added as a source of non-specific antibodies. CD16-transduced NK92 cells were added at an effector-to-target ratio of 3:1 before incubation for 180 min at 37 °C. Cells were washed and resuspended in PBS + 0.2% BSA + D-luciferin. Bioluminescence was measured after 25 min at 37 °C and the degree of ADCC induction was calculated as described above.

### CDC of primary myeloma cells

The studies involving human participants were approved by the ethics committee of Ärztekammer Hamburg (PV5505) and were carried out in adherence to the Declaration of Helsinki. All patients provided written informed consent.

Primary myeloma cells were obtained from bone marrow aspirates of myeloma patients. Bone marrow mononuclear cells (BM-MNCs) were isolated by Ficoll-Paque density gradient centrifugation (Sigma-Aldrich, St. Louis, MO, USA). Erythrocytes were depleted by incubation in red blood cell lysis buffer. BM-MNCs were stained for flow cytometric analysis using a panel of fluorochrome-conjugated antibodies (CD19 (clone HIB19, BD, cat. #555,414), CD38 (clone JK2^62^), CD56 (clone B159, BD, cat. #560,916), CD138 (clone MI15, BioLegend, cat. #356,516), CD229 (clone hLy9.1.25, BioLegend, cat. #326,108), CD269 (clone 19F2, BioLegend, cat. #357,508), and CD319 (clone 162.1, BioLegend, cat. #331,816)). Multiple myeloma cells were identified by high expression of CD38 and CD138. Viability was assessed using the amine reactive Pacific Orange succinimidyl ester (cat. #P30253, Thermo Fisher, Waltham, MA, USA)^31^.

For CDC assays, BM-MNCs were incubated in PBS + 0.2% BSA containing ~ 100 nM BAR or 100 nM daratumumab. When two BARs were combined, 50 nM of each construct was used. 25% human pooled serum was used as a source of complement and non-specific antibodies and cells were incubated at 37 °C for 90 min. Cells were washed once and stained for flow cytometry using fluorochrome-conjugated antibodies specific for CD38 and CD138. Staining of CD38 in the presence of CD38-BARs was achieved using an AlexaFluor 647-conjugated nanobody (clones WF211, MU1067, or JK36) that binds CD38 independently of the nanobody used in the CD38-BAR. Myeloma cell counts were quantified using CountBright absolute counting beads (Invitrogen, Waltham, MA, USA). Flow cytometric data was analyzed using FlowJo v10 (Becton Dickinson). The degree of CDC induction was determined as follows:

$$\% {\text{ live}}\;{\text{cells}} = \frac{{{\text{MM}}\;{\text{cell}}\;{\text{count}}\;{\text{per}}\;\upmu {\text{L}}\; [{\text{sample}}]}}{{{\text{MM}}\;{\text{cell}}\;{\text{count}}\;{\text{per}}\;\upmu {\text{L}}\;{\text{[ctrl-BAR]}}}} \times 100 \%$$

### In vivo and ex vivo imaging

All in vivo experiments were approved by the animal welfare commission (Behörde für Justiz und Verbraucherschutz, TVA N015-2023) and were carried out in accordance with national regulations and the ARRIVE guidelines. Five-weeks old, female NMRI-Foxn1nu mice were obtained from Charles River Laboratories (Sulzfeld, Germany) and allowed to adapt to their environment for two weeks before the start of the experiment. Animals were scored daily according to a pre-defined scoring sheet approved by the animal welfare commission. Prior to optical in vivo imaging, animals were kept on an alfalfa-free diet for 7 days to reduce fluorescence of the intestine. The average weight of the mice was 25.04 ± 1.97 g.

Mice were randomly assigned to two treatment groups (BAR only, n = 4 or BAR + IgG, n = 4), anesthetized with 2.5% isoflurane and baseline fluorescent images for the 0 h time point were acquired from the ventral and dorsal position (IVIS-200, PerkinElmer). Mice in the BAR + IgG group were then injected with 2 mg daratumumab in 100 µL of isotonic saline via the tail vein to provide a source of human IgGκ. 1 h post injection of daratumumab, all mice in both treatment groups were injected with 50 µg E3-BAR conjugated to the near-infrared fluorescent dye Alexa Fluor 680. Fluorescent images were acquired at 1, 2, 3, 6, 12, and 24 h after injection of the BAR.

At the 24 h time point, mice were euthanized by cervical dislocation under deep isoflurane anesthesia. Organs were explanted and their fluorescence was recorded using the IVIS-200 imaging device. Serum was removed and used to stain LP-1 luc cells at a dilution of 1:5 (v/v) in PBS + 0.2% BSA for 30 min at 4 °C. Cells stained in vitro with 1:100 (v/v) AF680-conjugated E3-BAR served as positive control, unstained cells served as negative control. Fluorescence was measured using a FACS Canto II (Becton Dickinson) and analyzed in FlowJo v10 (Becton Dickinson).

Fluorescent images were analyzed in Living Image 4.2 software (PerkinElmer). Rectangular regions of interest (ROIs) were drawn around each mouse to determine dorsal whole-body fluorescence as described previously^75^. Average radiant efficiencies [(p/sec/cm^2^/sr)/(µW/cm^2^)] were determined as a measure of remaining BAR. Data was further analyzed in GraphPad Prism 10.2.3 (GraphPad) to determine half-life. To this end, average radiant efficiencies were normalized to the 1 h time point, set to 100%. A non-linear regression using one-phase decay was performed to approximate whole-body half-life. Y0 was unconstrained, plateau was constrained to 0, K was constrained to > 0.

### Statistical analysis

Data were analyzed using GraphPad Prism 10.2.3 (GraphPad) and are presented as means ± standard deviation (SD). A normal data distribution was assumed. For cell culture and primary myeloma cell analyses, a one-way ANOVA followed by Dunnett’s multiple comparison test was used to determine statistically significant differences between the ctrl-BAR versus CD38-specific BARs (CDC and ADCC) or serum versus CD38-specific BARs (hemolysis assay). Nonlinear regression using a four-parameter logistic (4PL) model was performed on data normalized to the ctrl-BAR to determine EC_50_ values for in vitro CDC. For ex vivo analyses, a two-way ANOVA followed by Šídák’s multiple comparison test was used to detect differences between the same organs from the BAR only versus the BAR + IgG group. The difference in LP-1 staining using serum from the BAR only group versus the BAR + IgG group was compared by a two-tailed Welch’s t-test. *P* < 0.05 was considered significant for all tests.

## Supplementary Information

Supplementary Information 1.

Supplementary Information 2.

## Data Availability

The data that support the findings of this study are available from the corresponding author upon reasonable request.
