# Supplementary material for: CD38-specific nanobody-based bispecific antibody recruiters (BARs) redirect complement-dependent cytotoxicity toward multiple myeloma cells
Source: Sci Rep. 2025 Nov 18;15:40376. doi: 10.1038/s41598-025-25194-y (PMC12627525; doi:10.1038/s41598-025-25194-y)
Supplement: Supplementary file 1 — Supplementary Information 1. [file 41598_2025_25194_MOESM1_ESM.pdf]

Supplementary Information

CD38-specific nanobody-based bispecific antibody recruiters (BARs) redirect complement-dependent cytotoxicity toward multiple myeloma cells

*Luca Julius Pape[1,2], Anna Josephine Gebhardt[1], Marten Dannenberg[1], Henry Risch[1], Anya Duttmann[1,2], Katja Weisel[3], Julia Hambach[1,2], Friedrich Koch-Nolte[2], Peter Bannas[1]**

[1]Department of Diagnostic and Interventional Radiology and Nuclear Medicine, University Medical Center Hamburg-Eppendorf, Hamburg, Germany

[2]Institute of Immunology, University Medical Center Hamburg-Eppendorf, Hamburg, Germany

[3]Department of Oncology, Hematology and Bone Marrow Transplantation, University Medical Center Hamburg-Eppendorf, Hamburg, Germany

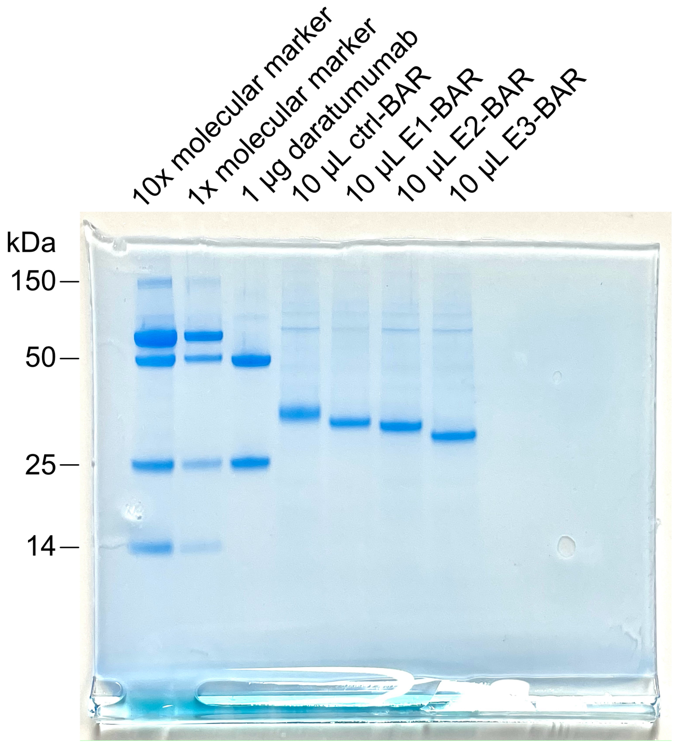

Supplementary Figure 1: Coomassie staining of an SDS PAGE run under reducing conditions with HEK cell supernatants of BARs and purified daratumumab.

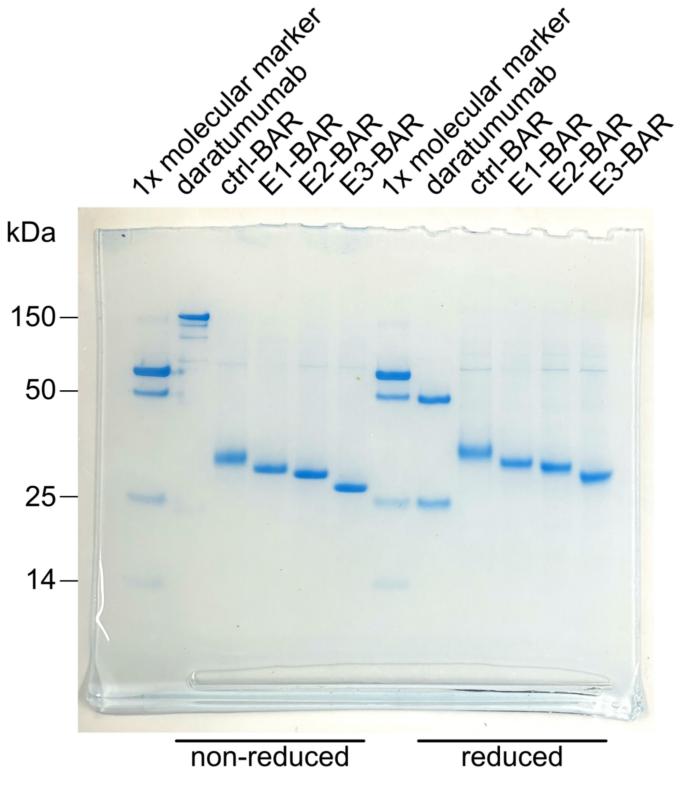

Supplementary Figure 2: Coomassie staining of an SDS PAGE with 10 µL HEK cell supernatants of CD38-BARs and 1 µg purified daratumumab run under non-reducing and reducing conditions.

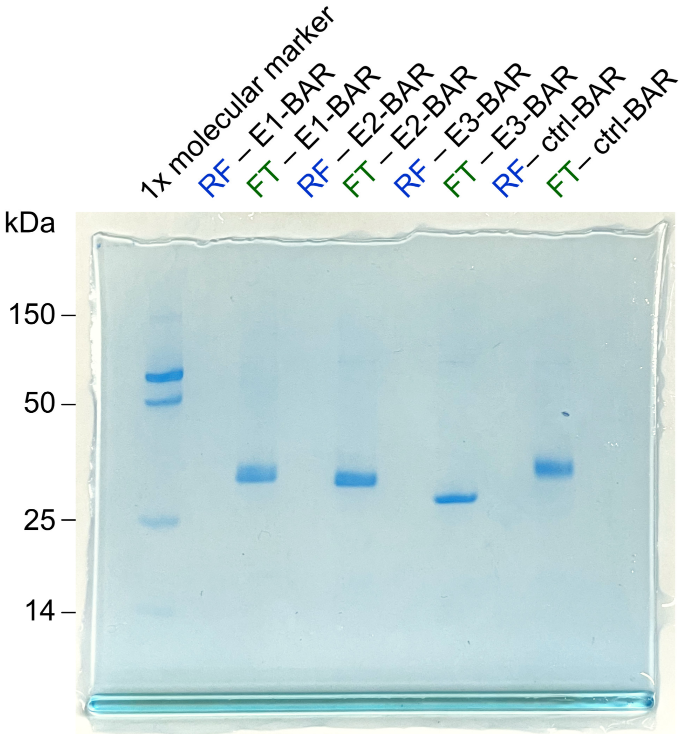

Supplementary Figure 3: Coomassie staining of a non-reducing SDS PAGE with 10 µL HEK cell supernatants of CD38-BARs and ctrl-BAR. To assess protein aggregation, samples were centrifuged using Amicon Ultra centrifugal filters with a 100 kDa cutoff and retained fraction (RF) and flow-through (FT) were compared.

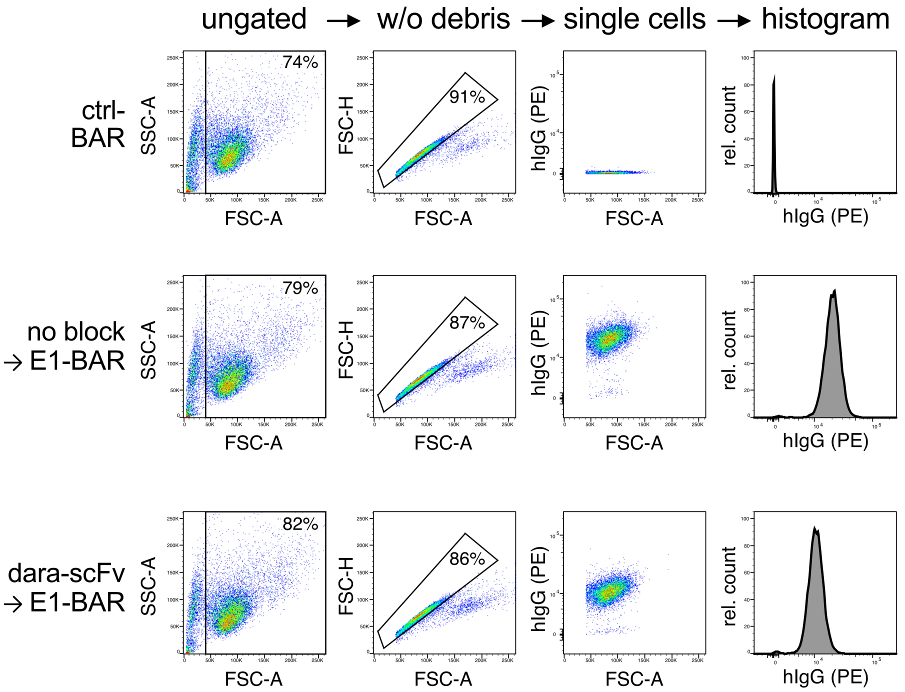

Supplementary Figure 4: Gating strategy for flow cytometry data in Fig. 5 using three exemplary samples. CD38-expressing HEK cells were blocked with or without daratumumab-scFv. BARs were then allowed to associate and inactivated human serum was added as a source of IgG antibodies. Human IgG was stained using a PE-conjugated secondary antibody. An FSC threshold was set to exclude debris. Gating was then performed on single cells. Percentage of included cells is indicated in each gate.

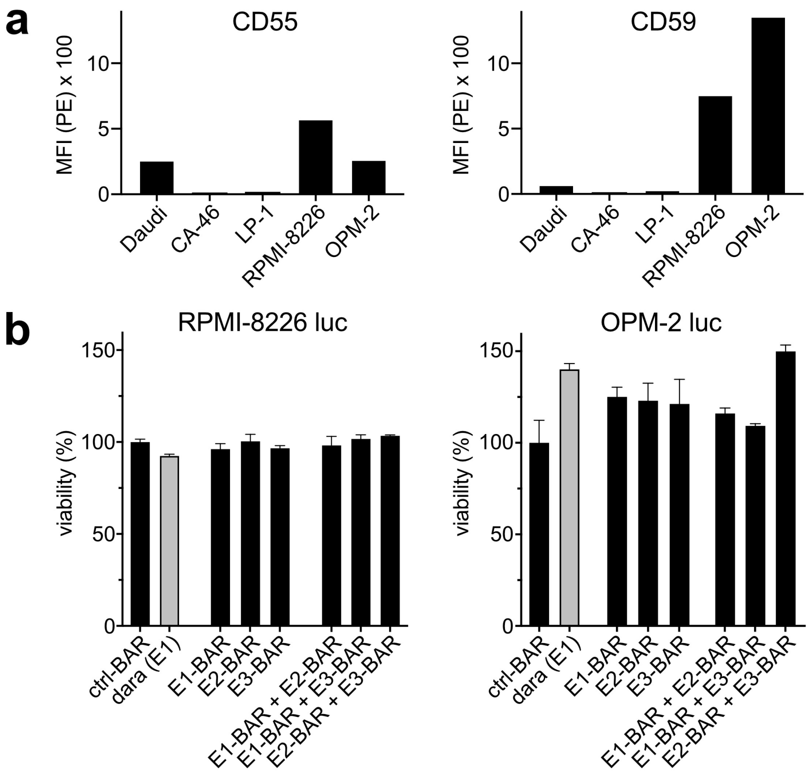

Supplementary Figure 5: a) Median fluorescent intensity (MFI) of complement-inhibitory proteins CD55 and CD59 was determined by flow cytometry for CA-46, Daudi, LP-1, RPMI-8226, and OPM-2 cells. **b)** Complement-dependent cytotoxicity assay in RPMI-8226 and OPM-2, the cell lines with the highest expression of CD55 and CD59.
